# 2’-Fucosyllactose inhibits proliferation of *Clostridioides difficile* ATCC 43599 in the CDi-screen, an *in vitro* model simulating *Clostridioides difficile* infection

**DOI:** 10.3389/fcimb.2022.991150

**Published:** 2022-10-28

**Authors:** Maria Wiese, Frank H. J. Schuren, Wiep Klaas Smits, Ed J. Kuijper, Anita Ouwens, Margreet Heerikhuisen, Louise Vigsnaes, Tim J. van den Broek, Paulo de Boer, Roy C. Montijn, Jos M. B. M. van der Vossen

**Affiliations:** ^1^ Microbiology and Systems Biology, The Netherlands Organization for Applied Scientific Research (TNO), Leiden, Netherlands; ^2^ Department of Medical Microbiology, Leiden University Medical Center, Leiden, Netherlands; ^3^ Glycom A/S—DSM Nutritional Products Ltd., Kogle Allé 4, Hørsholm, Denmark; ^4^ Department of Technology, Faculty of Health, University College Copenhagen, Copenhagen, Denmark

**Keywords:** *Clostridioides difficile* infection, *in vitro* gut model, screening, interventions, human milk oligosaccharide

## Abstract

**Background:**

*Clostridioides difficile* is a Gram-positive anaerobic bacterium that can produce the toxins TcdA and/or TcdB and is considered an opportunistic pathogen. *C. difficile* is mainly transmitted as endospores, which germinate to produce the pathogenic vegetative cells under suitable conditions in the gut. To efficiently screen novel therapeutic- interventions against the proliferation of *C. difficile* within a complex microbial community, platforms are needed that facilitate parallel experimentation. In order to allow for screening of novel interventions a medium-to-high throughput *in vitro* system is desirable. To this end, we have developed the 96-well CDi-screen platform that employs an adapted simulated ileal effluent medium (CDi-SIEM) and allows for culturing of pathogenic *C. difficile*.

**Methods:**

*C. difficile* strain ATCC 43599 was inoculated in the form of vegetative cells and spores into the CDi-screen in the presence and absence of a cultured fecal microbiota and incubated for 48h. To demonstrate its utility, we investigated the effect of the human milk oligosaccharide 2’-Fucosyllactose (2’-FL) at 4 and 8 mg/mL on *C. difficile* outgrowth and toxin production in the CDi-screen. The test conditions were sampled after 24 and 48 hours. *C. difficile* -specific primers were used to monitor *C. difficile* growth *via* qPCR and barcoded 16S rRNA gene amplicon sequencing facilitated the in-depth analysis of gut microbial community dynamics.

**Results:**

*C. difficile* ATCC 43599 proliferated in CDi-SIEM, both when inoculated as spores and as vegetative cells. The strain reached cell numbers expressed as *C. difficile* genome equivalents of up to 10 ^8^ cells per mL after 24h of incubation. 2’-FL significantly inhibited the outgrowth of the ATTC 43599 strain within a complex human gut microbial community in the CDi-screen. In addition, a dose-dependent modulation of the gut microbial community composition by 2’-FL supplementation was detected, with a significant increase in the relative abundance of the genus *Blautia* in the presence of 2’-FL.

**Conclusion:**

The CDi-screen is suitable for studying *C. difficile* proliferation in a complex gut ecosystem and for screening for anti-pathogenic interventions that target *C. difficile* directly and/or indirectly through interactions with the gut microbiota. Different doses of compounds such as in this study the dose of the human milk oligosaccharide 2’-FL can be screened for efficacy in the inhibition of *C. difficile* proliferation.

## Introduction

*Clostridioides difficile* (Lawson et al., 2016; Oren and Rupnik, 2018), is a spore-forming Gram-positive bacterium that poses a severe threat to human health. Many *C. difficile* strains produce two high molecular weight toxins, toxin A and toxin B (Smits et al., 2016). In addition, some strains produce a binary toxin, also contributing to pathogenesis (Bartlett, 2002). The toxins trigger a complex cascade of host cellular responses to cause diarrhea, inflammation, and tissue necrosis — the main symptoms of *C. difficile* infection (CDI) (Smits et al., 2016). The toxin regulatory network overlaps with sporulation, motility, and key metabolic pathways (Majumdar and Govind, 2022).

Another factor contributing to the pathogenesis of *C. difficile* is it’s ability to form spores. Dormant spores allow *C. difficile* to survive for prolonged periods in extreme conditions (Zhu et al., 2018). Germination of *C. difficile* spores is stimulated by elevated levels of certain bile acids (e.g., taurocholic acid) combined with amino acids as co-germinants (Baloh and Sorg, 2022). A disturbed balance of bile acids is often associated with the germination of spores and the onset of disease (Crowther et al., 2016; Kazakova et al., 2020; Qian et al., 2020). CDI is considered the leading cause of antibiotic-associated diarrhea in hospitals (Bartlett, 2002; Guh and Kutty, 2018), and is now also increasingly recognized as a community-acquired disease (Khanna et al., 2012; Hensgens et al., 2014), accounting for up to 41% of the total CDI cases reported (Penit et al., 2016). In 2016, an estimated 125.000 patients and at least 6.000 deaths related to CDI were reported in Europe (Smits et al., 2016).

Approved anti-CDI treatments are still primarily based on specific antibiotics such as metronidazole, vancomycin, and fidaxomicin (Johnson et al., 2021). Nevertheless, recurrence of CDI is observed after antibiotic treatment in 15-35% of the cases (Leffler and Lamont, 2015). Other approved treatments include bezlotoxumab, a monoclonal antibody neutralizing the TcdB toxin, and fecal microbial transplantation (FMT) (Khanna et al., 2012; Baunwall et al., 2021) and other microbiota-based interventions are on the horizon (Feuerstadt et al., 2022; Klein, 2022).

More advanced and refined treatments are likely to be a preferable solution against enteropathogenic Clostridia in the future. Therefore, it is pivotal to develop models that facilitate the study of *C. difficile* biology and anti-*C. difficile* interventions *in vitro*. A few *C. difficile in vitro* models exist; some models operate at a relatively large volume e.g., the Pathogut™ model (specific application of the Simulator of the Human Intestinal Microbial Ecosystem (SHIME^®^) (Crowther et al., 2016; Vigsnaes et al., 2021).

The anti-pathogenic effects of human milk oligosaccharides (HMOs) against *C. difficile* ATCC 9689 were reported based on Pathogut experiments. The effects coincided with increased levels of *Bifidobacteriaceae* and/or secondary bile acids (Vigsnaes et al., 2021).

Nevertheless, the large volume, low throughput, and related experimental designs are usually compromising experimental replicates and do not facilitate the screening of novel and expensive compounds available in small amounts.

Microbiota-mediated effects such as the protective aspects of the commensal carbohydrate metabolism suggest novel avenues to develop probiotic or prebiotic therapeutics against CDI (Fishbein et al., 2021). Non-digestible oligosaccharides and short-chain fatty acids (SCFA) have been described as therapeutic targets against enterotoxin-producing bacteria (Asadpoor et al., 2021). Together, these findings underscore the potential contribution of *in vitro* studies for the research and development of novel interventions and therapies against CDI, that target *C. difficile* directly or through microbiota-mediated effects. *In vitro* gut model studies can facilitate the functional screening of pre- and probiotics regarding their modulatory effects on the gut-microbial community dynamics and relevant metabolites such as SCFA and secondary bile acids. In the context of CDI research, several studies implementing *in vitro* model systems, in some cases relying on (mini) bioreactors, have been described (Auchtung et al., 2016; Crowther et al., 2016; Fehlbaum et al., 2016; Horvat and Rupnik, 2018). Nevertheless, there is still a need and potential for developing screening systems operating with an even smaller volume to facilitate more time- and cost-effective screening of novel interventions against enteropathogenic Clostridia.

In the current study, a 96-deep-well microtiter plate-based fermentation system is presented for studying *C. difficile* biology, anti-*C. difficile* interventions, and microbial interactions in cultivated human gut microbiota. This *C. difficile*-i-screen (CDi-screen) is based on the i-screen (Ladirat et al., 2013; Schuren et al., 2019) and was optimized for germination and outgrowth of *C. difficile* spores and vegetative cells. This *in vitro* platform allows for the screening of several variables (e.g. vegetative cell and spore load) in replicates. Direct and gut microbiota-mediated inhibitory effects on *C. difficile* of different interventions can be investigated in just 1 mL of culture volume per replicate, facilitating the acquisition of insights with statistical relevance largely compromised when working with *in vitro* models that operate at a larger volume and with low throughput.

## Materials and methods

### Bacterial strain

*C. difficile* strain ATCC 43599 was used for the experiments. This strain, originally isolated from human fecal material in Belgium (Lemee et al., 2004), belongs to Serogroup G, is *tcdA* and *tcdB* positive (Lemee et al., 2004), has Toxinotype 0, and falls within polymerase chain reaction (PCR) ribotype 001 (Clade 1), which is one of the most abundant ribotypes in Europe (Bauer et al., 2011; Freeman et al., 2020).

### Routine cultivation of *C. difficile*

*C. difficile* was cultivated from frozen glycerol stock on Schaedler’s Anaerobic Agar (SAA) plates containing 0.2% sodium taurocholate hydrate (Sigma Aldrich Chemie NV, Netherlands, 86339) for 48 h at 37°C under anaerobic conditions in an A45 anaerobic workstation (Don Whitley Scientific Ltd, UK) using a gas mixture of 80% nitrogen, 10% carbon dioxide and 10% hydrogen. Liquid cultures were prepared in Brain Heart Infusion medium (BHI; Oxoid) and incubated for 20 h at 37°C in an A45 anaerobic workstation. *C. difficile* was cultured in BHI supplemented with 0.25% (w/v) fructose (Edwards and McBride, 2016) and microscopically checked for the presence of >99% vegetative cells, which were then used as inoculum for experiments.

### *C. difficile* sporulation and harvesting of spores

Spores of the *C. difficile* strain were prepared by culturing *C. difficile* on 70:30 sporulation plate medium (Edwards and McBride, 2016). Inoculated plates were incubated for 15 days in the anaerobic chamber at 37°C. The number of mature spores was determined by phase-contrast microscopy, where they show up as phase-bright. The spores were harvested from the plates as described (Edwards and McBride, 2016). The spore suspension was incubated at 70°C for 20 minutes to inactivate any residual viable vegetative cells. Viable spore counts were determined by seeding serial dilutions on SAA agar medium (Oxoid) supplemented with 0.2% sodium taurocholate hydrate. SAA agar medium was also used for enumerating viable cells of the *C. difficile* cultures.

### Pure-culture cultivation in CDi-SIEM

To allow *C. difficile* to germinate, grow and produce toxins in the presence of human gut microbiota *in vitro*, a suitable culture medium was formulated, which was named CDi-SIEM. CDi-SIEM contained per litre: 4.5 g NaCl, 2.5 g K_2_HPO_4_, 0.45 g CaCl_2_·2H_2_O, 0.4 g MgSO_4_·7H_2_O, 0.01 g FeSO_4_·7H_2_O, 0.4 g ox bile, 0.01 g haemin, 0.05 g pectin, 0.05 g xylan, 0.05 g arabinogalactan, 0.05 g amylopectin, 0.4 g starch, 24 g bactopeptone, 24 g casein, 10 mL 1M MES buffer pH 6.0 and 0.8 mL of a vitamin mixture containing per litre: 1 mg menadione, 2 mg D-biotin, 0.5 mg vitamin B-12, 10 mg pantothenate, 5 mg nicotinamide, 5 mg para-aminobenzoic acid, and 4 mg thiamine. All medium components were provided by Tritium Microbiology (Veldhoven, the Netherlands). The final pH of the medium was 6.0. Where appropriate, CDi-SIEM was supplemented with 0.2% sodium taurocholate hydrate to stimulate the germination of *C. difficile* spores (Sorg and Sonenshein, 2010).

Preparation of vegetative cells as inoculum for pure *C. difficile* culture experiments in CDi-SIEM was done by first preparing an overnight (o/n) culture from an SAA plate in CDi-SIEM under anaerobic conditions at 37°C. This pre-culture was 10 x diluted in peptone physiological salt solution and subsequently diluted 100 x in CDi-SIEM as start inoculum, resulting in ~10^5^ colony forming units (CFU) mL^-1^. The number of vegetative cells used as inoculum for the CDi-screen was adjusted based on experience with optical density measurements at 600 nm, an estimated *C. difficile* cell number was spiked into the complex microbiota followed by plate counting for retrospective confirmation. The spore numbers in the spore inoculum were determined by plate counting before the experiments, the spore numbers remained stable in the inoculum suspension. The inoculation level of *C. difficile* cells and spores in the CDi-screen is based on experience with spiking of a bacterial species into an active gut microbiota culture to conclude on growth capacity of the strain and also allow for a growth range to evaluate interventions. Alternatively, *C. difficile* spores from a spore stock were added as inoculum to a final concentration of 10^5^ CFU mL-1 in CDi-SIEM.

### Cultivation of *C. difficile* in the CDi-screen

To study *C. difficile* growth in the context of a microbial human gut community, fecal microbiota were collected from 4 healthy European adults (37–64 years old). These individuals neither received antibiotic treatments 3 months before donation nor consumed prebiotics or probiotics the week before donation. The individual fecal donations were placed under anaerobic conditions as soon as possible and processed within 24 h under anaerobic conditions. Processing consisted of preparing fecal slurries consisting of 1 part fecal material, 4 parts of pre-reduced standard ileal effluent medium (Minekus et al., 1999; Ladirat et al., 2013), and 1 part pre-reduced sterile 87% glycerol. A pool was made from these individual slurries by mixing equal quantities of each fecal pool, and 0.5 mL aliquots of this pooled material were stored at -80°C as inoculum for the CDi-screen.

For starting a CDi-screen experiment, 400 µl of a frozen aliquot of the fecal pool was pre-cultured o/n in 10 mL CDi-SIEM medium under anaerobic conditions (37°C; 300 RPM). This o/n pre-culture of the fecal microbiota was mixed as a 100x dilution with pre-reduced CDi-SIEM*. C. difficile* was added to this suspension, similar to inoculation of pure culture *C. difficile* cultivations in CDi-SIEM. Finally, the suspension was distributed as 1.5 mL aliquots into the wells of a 96-deep-well plate (Axygen P-DW-20C). The inoculated plates were then sealed with gas permeable seals and incubated at 37°C at 300 RPM under anaerobic conditions for 48 h. After 24, and 48 h incubation, a 50 µl sample was harvested into a fresh deep-well plate for DNA extraction and subsequent molecular analyses and a 200 µl sample was harvested for toxin measurement.

### Total DNA extraction from cultures in 96 deep-well plates

Each well of a 96-deep-well plate containing 50 µL culture was supplemented with 500 µL washed zirconium beads (0.1mm; BioSpec products, Bartlesville, USA) in Milli-Q water and 800 µL CD1 solution from the DNeasy 96 Powersoil Pro QIAcube HT Kit (Qiagen). The plate was sealed and homogenized 2 x 2 min in a run. Subsequently, the plates were centrifuged at 3000 ×g for 6 min. Then, 600 µL of supernatant was transferred into a fresh plate containing 300 µL CD2 solution from the DNeasy 96 Powersoil Pro QIAcube HT Kit (Qiagen) and mixed by pipetting up and down three times. After centrifugation of the plate at 3000 × g for 6 min, 550 µL of the supernatant was transferred to an S-block (Qiagen). From this point on, the DNA was purified according to the Qiacube protocol (Qiagen). Purified DNA was stored at -20°C in EB buffer (Qiagen).

### Quantification of *C. difficile* by qPCR

The presence and abundance of *C. difficile* were analyzed by a *C. difficile*-specific 16S quantitative polymerase chain reaction PCR (qPCR) on DNA isolated from cultures at different time points. For the analysis of each sample, a 25 µL PCR reaction mixture was prepared to contain 0.5 µL of DNA sample (10 pg to 1 ng), 12.5 µL 2x Diagenode Master Mix (Diagenode, Seraing, Belgium), 0.2 µM probe (VIC -5’-TGACATCCCAATGACA-3’-MGB), 0.4 µM forward primer (5’-GCAACGCGAAGAACCTTACCTA-3’), and 0.4 µM reverse primer (5’-GAAGGGAACTCTCCGATTAAGGA-3’). An Applied Biosystems 7500 FastReal-Time PCR system (Foster City, CA, USA) was used for qPCR with temperature cycle settings: 2 minutes 50°C, 10 min at 95°C, 35 cycles with alternating 15 sec at 95°C, and 60 sec. at 60°C.

### Microbiota composition analysis by 16S rDNA gene amplicon sequencing

Microbiota composition was analyzed by 16S rDNA amplicon sequencing of the V4 hypervariable region. An estimation of the amount of amplifiable bacterial 16S rDNA in each sample of the CDi-screen was obtained by qPCR using universal primers targeting the bacterial 16S rRNA gene irrespective of the taxonomic position (16Suni-I-F 5’CGA AAG CGT GGG GAG CAA A 3’, 16Suni-I-R 5’GTT CGT ACT CCC CAG GCG G 3’ and 16Suni-I 5’ATT AGA TAC CCT GGT AGT CCA 3’ FAM -MBG probe). This qPCR was executed as described in the previous paragraph. Based on the DNA quantity in the individual samples, 1 ng of DNA of each sample was used to generate PCR amplicons of the V4 hypervariable region of the 16S rRNA gene as described before (Kozich et al., 2013), using the F515/R806 primers (Caporaso et al., 2011). Primers included Illumina adapters and a unique 8-nt sample index sequence key (Kozich et al., 2013). Amplified DNA was quantified using the dsDNA 910 Reagent Kit on a Fragment Analyzer (Advanced Analytical). The amplicon libraries were pooled in equimolar amounts and purified from 1.2% agarose gel using the Gel Extraction Kit (Qiagen). The library was quantified using the Quant-iT™ PicoGreen^®^ dsDNA Assay Kit (Thermo Fisher Scientific). Paired-end sequencing of amplicons was conducted on an Illumina MiSeq platform (Illumina, Eindhoven, The Netherlands). The sequencing data pre-processing, analysis, and classifications were performed using modules implemented in the Mothur software platform (Khanna et al., 2012). Chimeric sequences were identified and removed using the chimera.uchime command. Unique 16S rDNA sequences were aligned using the align.seqs command and the Mothur-compatible Bacterial SILVA SEED database (Release 119; available online: https://mothur.org/wiki/Silva_reference_files). Bacterial sequences were taxonomically classified by the RDP-II Naïve Bayesian Classifier using a 60% confidence threshold against the RDP Database (Release 11.1; available online: https://www.mothur.org/wiki/RDP_reference_files) for 16S rDNA.

Raw sequence data, including metadata, are available through the European Nucleotide Archive. Temporary SubmissionID: SUB11651667.

### Statistical analysis

For the qPCR data two linear models were created on log10 transformed data using the lm function from R version 4.1.2, from which all statistical results were subsequently derived, with one model for each type of inoculum (Team, 2021a). P-values for the post-hoc tests were calculated using the emmeans package (Lenth et al., 2021). No multiple testing correction was applied. P-values <0.05 were deemed statistically significant. The ggplot2 package was used for data visualization (Wickham, 2016). For the sequencing data all statistical analyses were performed using R version 4.1.2 (Team, 2021b). All figures were composed using the ggplot2 package version 3.3.5 (Wickham, 2016).

Multivariate analysis and ordinations were performed using the vegan package, version 2.5-7 (Oksanen et al., 2021). This package was also used to calculate the inverse Simpson alpha-diversity.

The multivariate models fitted by PERMANOVA were tested by permutation analysis to produce Type III (marginal) p-values for the terms included in the model. 10^3^ permutations were used for all reported results. Count data were transformed using the center-log ratio transformation in the case of PERMANOVA, RDA, and PCA ordinations. This was done using the compositions package, version 2.0-2 (Van den Boogaart et al., 2021).

The 16S data were filtered to include only those taxa that contribute to the first 97.5% of all counts in the data. This step was not performed for the alpha-diversity analysis. The filtering procedure consisted of the steps denoted below. Given the count table of taxa A, where A is an i,j matrix (samples by taxa), let the row normalized matrix be denoted by D, where D is an i,j matrix, with the formula given by


$$D_{i,j}=\frac{A_{i,j}}{\sum_{\theta =1}^{n}A_{i,\theta }}$$

let the row count normalized column sums of our matrix D be denoted as the vector c, given by


$${\hat{c}}_{j}=\;\sum_{\theta =1}^{m}D_{\theta ,j}\forall j\in [1,n]$$

the cumulative sum of the column sums normalized for column count is then denoted as C where


$$c=\;\sum_{\theta =1}^{n}\frac{{\hat{c}}_{\theta }}{n}$$

any taxa where C is smaller than 0.975 (corresponding to 97.5%) was then chosen to be included in the analysis. This procedure eliminates sparse, low-count taxa from the dataset.

### Biostatistical analysis of conditions affecting *C. difficile* growth and toxin production in complex human fecal microbiota

Multidimensional scaling (MDS) plots, were used to visualize similarities and distances between samples and groups of samples, including *C. difficile* growth and toxin production. MDS is a set of ordination techniques that allows the translation of information about the pairwise distances among a set of objects (in this case, the different samples and replicates) into a configuration of points in a two-dimensional space, thereby visualizing differences and similarities between the objects in terms of their relative position in the space.

### Toxin measurements

Samples were thawed at room temperature and filtered with GE Healthcare Unifilter microplate devices to separate solid cell matter from the supernatant. In brief, after sample loading, filter plates with samples were centrifuged for 5 minutes at 4000 rpm setting in Eppendorf Centrifuge 5810 R at 4. The remaining supernatant was used for toxin analysis. The *C. DIFFICILE TOX A/B II ™* ELISA Kit (TechLab, Blacksburg, VA, USA) was used to determine the toxin content in the sample. Instead of the recommended dilution in a diluent according to the manufacturer’s instructions for liquid fecal samples, 100 µl of undiluted sample was analyzed. The *in vitro* sample supernatant is more liquid than conventional feces and additional dilution was hence avoided. According to the manufacturer, optical density (OD) 450 nm below 0.12 is inconclusive/below the detection limit, and “the C. DIFFICILE TOX A/B II™ test will detect Toxin A at levels ≥0.8 ng/mL and Toxin B at levels ≥2.5 ng/mL. ODs were measured with a Envision 2105 Multimode Plate Reader (PerkinElmer).

## Results

### CDi-SIEM sustains the growth of *C. difficile* ATTC 43599

We have assessed whether the levels of germinants were sufficient for the germination of spores of the ATTC 43599 strain and result in bacterial growth, both in the absence and presence of additional supplementation of with 0.2% taurocholate.

The proliferation of the ATTC 43599 strain (vegetative cells and spores) in the CDi-SIEM with and without supplementation of an additional 0.2% taurocholate at pH 6.0 throughout 48 h of incubation was investigated. Proliferation of *C. difficile* was quantified by qPCR using an in-house developed *C. difficile*-specific 16S qPCR, additionally we performed enumeration of colony forming units (CFU) based on dilution plating (**Supplementary Figure 1**).

When inoculated at a level of around 10^4^ (vegetative cells) or 10^5^ spores per mL, the ATTC 43599 strain reached a cell number of about 10^8^ per mL after 24 h of incubation as detected by qPCR and CFU counting.

The amount of germination-stimulating bile acid compounds in the ox bile added to the CDi-SIEM appears to be sufficient to support the germination of spores of the ATTC 43599 strain, and the additional taurocholate does not affect germination levels and cell counts. CFU counts and genome equivalents detected by qPCR correlate with drop-plate CFU counts for the sampling time points in the CDi-SIEM 24 h and 48 h when cultured in a deep-well plate. The CDi-SIEM medium supported toxin production above the limit of detection in monocultures after 48 h, when inoculated with vegetative cells in media supplemented with taurocholate (OD_450 nm_ 0.196), toxin measurements in the remaining conditions were below the detection limit (data not shown).

### *C. difficile* ATCC 43599 can proliferate in human fecal microbiota in the CDi-screen

We have inoculated the *in vitro* model with a complex microbial community and spiked the media with *C. difficile* spores or vegetative cells of the *C. difficile* strain ATCC 43599. This allowed us to assess the outgrowth of the ATCC strain within a complex microbial community after 24 h and 48 h of incubation. Furthermore, we supplemented the media with 4 and 8 mg/mL of the human milk oligosaccharide 2’FL to demonstrate the application of the CDi-screen for the testing of interventions against *C. difficile in vitro*.

**Figure 1:**
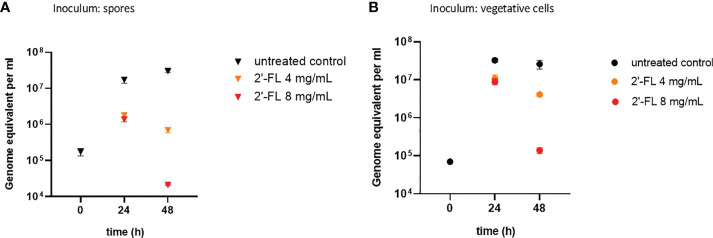
2'-FL inhibits proliferation of *C. difficile* in the CDi-screen model (panel **A** Inoculum: spore, panel **B** Inoculum: vegetative cells). Detection of *C. difficile* within a complex microbiota in the CDi-screen using *C. difficile* -specific 16S qPCR at t=0 and 24 h and 48 h, in the absence of 2’-FL (untreated control, black symbol), and media supplemented with 2’-FL at 4 mg/mL (orange symbol) and 8 mg/mL (red symbol). Data are plotted as genome equivalents per mL, derived from standard curves.

When inoculating the CDi-screen with spores, an increase in *C. difficile* abundance over time was detected within the CDi-screen experiments without 2'-FL supplementation, indicating that *in vitro* germination and proliferation of *C. difficile* occurred. *C. difficile* displayed growth to above 10^7^ genome equivalents per mL after 24 h of incubation, and cell numbers increased further after 48 h of incubation (**Figure 1**). Toxin levels were below the level of detection in the culture supernatant after 48 h, independent of 2'-FL supplementation (data not shown). When CDi-SIEM medium was supplemented with 2’-FL at 4 and 8mg/mL and inoculated with spores, just over 10^6^ C*. difficile* genome equivalents/mL were detected after 24 h of incubation, significantly less for both doses (4 and 8 mg/ml) (p<0.001) than in the condition without 2'-FL supplementation. This observation indicates an inhibitory effect of 2’-FL on *C. difficile* outgrowth. This effect was dose-dependent and more pronounced after 48 h of incubation when *C. difficile* cells decreased to just above 10^4^ genome equivalents per. The difference in *C. difficile* levels detected were significantly lower than in the untreated control (p<0.001) mL (**Figure 1A**).

Similar results were obtained when inoculating the complex human fecal microbiota with vegetative cells of the *C. difficile* ATTC 43599 strain: a dose-dependent inhibitory effect of 2'-FL on *C. difficile* proliferation was evident, in particular after 48h of incubation in comparison to the untreated controls (**Figure 1B**). The growth of *C. difficile* cells differed significantly in the 2'-FL supplemented media compared to the untreated control with a p-value small than 0.001 for both doses and both time points. Notably, we detected toxins at a level of OD_450nm_ 0.23 ± 0.06 in the control wells without 2’-FL, but toxin levels were below the limit of detection when *C. difficile* was grown in media supplemented with 2’-FL (data not shown).

### Microbial community composition in the CDi-screen model is reproducible and modulated by interventions

**Figure 2:**
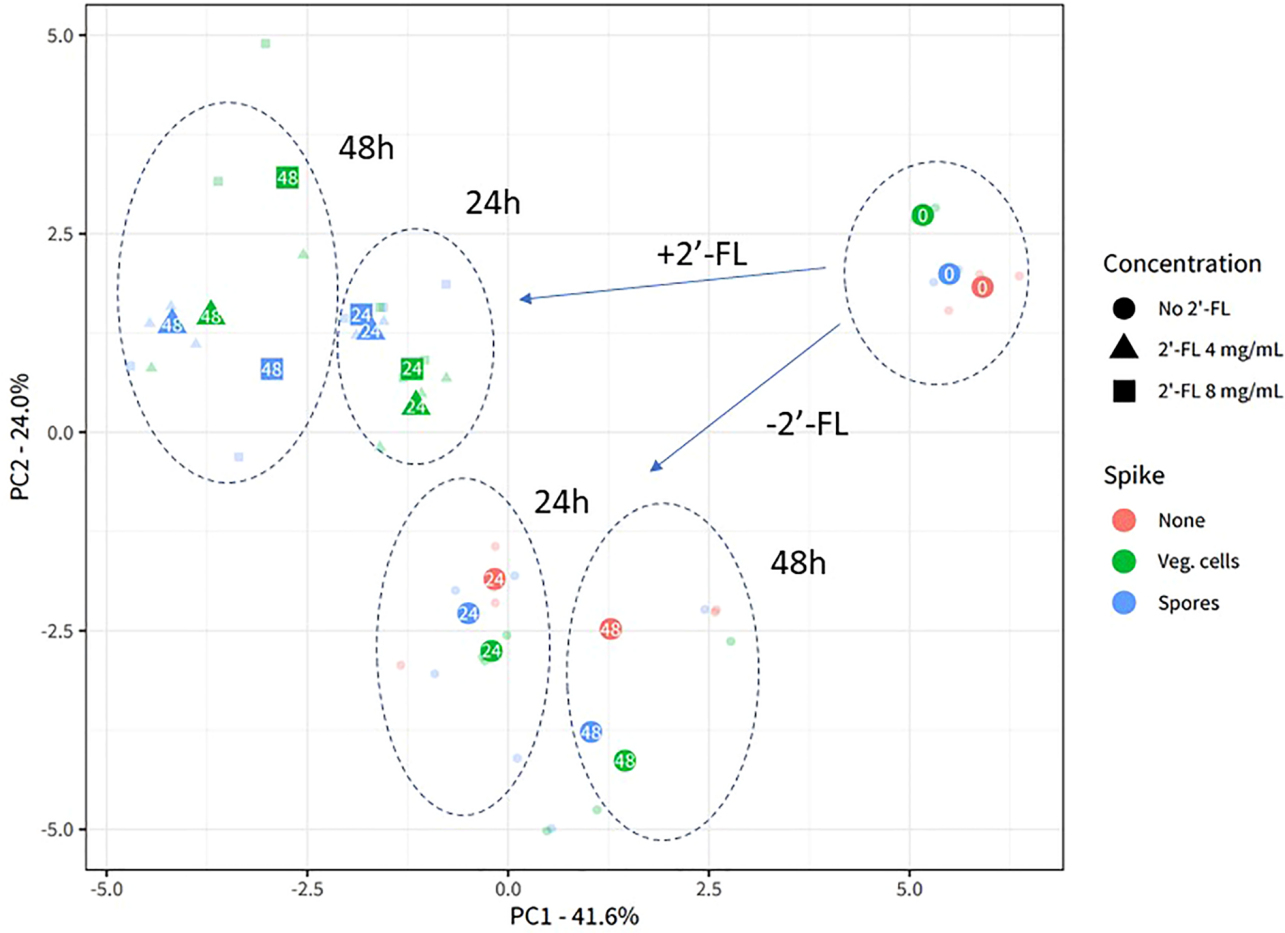
2’-FL affects 16S microbial community composition in the CDi-screen. Experimental reproducibility and differentiation of microbial communities across different time points and culture conditions in the CDi-screen are shown in the principal component analysis (PCA) ordination plot with center-log ratio transformed data. The fecal microbial community was analyzed without *C. difficile* (red) as well as with *C. difficile* vegetative cells (green) or spores (blue), and incubated in CDi-SIEM media at pH 6.0, without (circles) and with supplementation of the media with 2’-FL at doses of 4 mg/mL (triangles) and 8 mg/mL (squares), and sampled at t=0 h, 24 h, 48 h (indicated with numbers in the symbols). Only taxa that belong to the first 97.5% of all sequenced counts in the data are included. All experimental data with the relevant replicate condition is shown as the centroid of the three replicates for the condition, with each sample shown in lower opacity.

To assess the overall community composition in the CDi-screen model, we performed 16S rRNA amplicon sequencing. A principal component analysis was performed to provide an overview of the experimental reproducibility as well as to visualize the effects of the 2'-FL interventions on the microbial communities cultured in the CDi-screen *in vitro* (**Figure 2**).

The experimental replicates of each condition are displayed in lower opacity and clustered closely, demonstrating the experimental reproducibility of the CDi-screen. The sample clusters separate according to the sample time points and experimental conditions. Supplementation of the CDi-SIEM media with 2’-FL significantly changes microbial community composition compared to the no-compound incubations (Permanova statistics: Compound-effect R2 0.24, p-value 0.001, dose-effect R^2^ 0.27, p-value 0.001), with samples supplemented with 4 mg/mL 2’-FL clustering closely with samples supplemented with 8 mg/mL. Notably, the introduction of *C. difficile* into the microbial community does not appear to lead to a significant restructuring of the community, as the samples without *C. difficile* cluster closely with those inoculated with either vegetive cells or spores of *C. difficile* (**Figure 2**).

**Figure 3:**
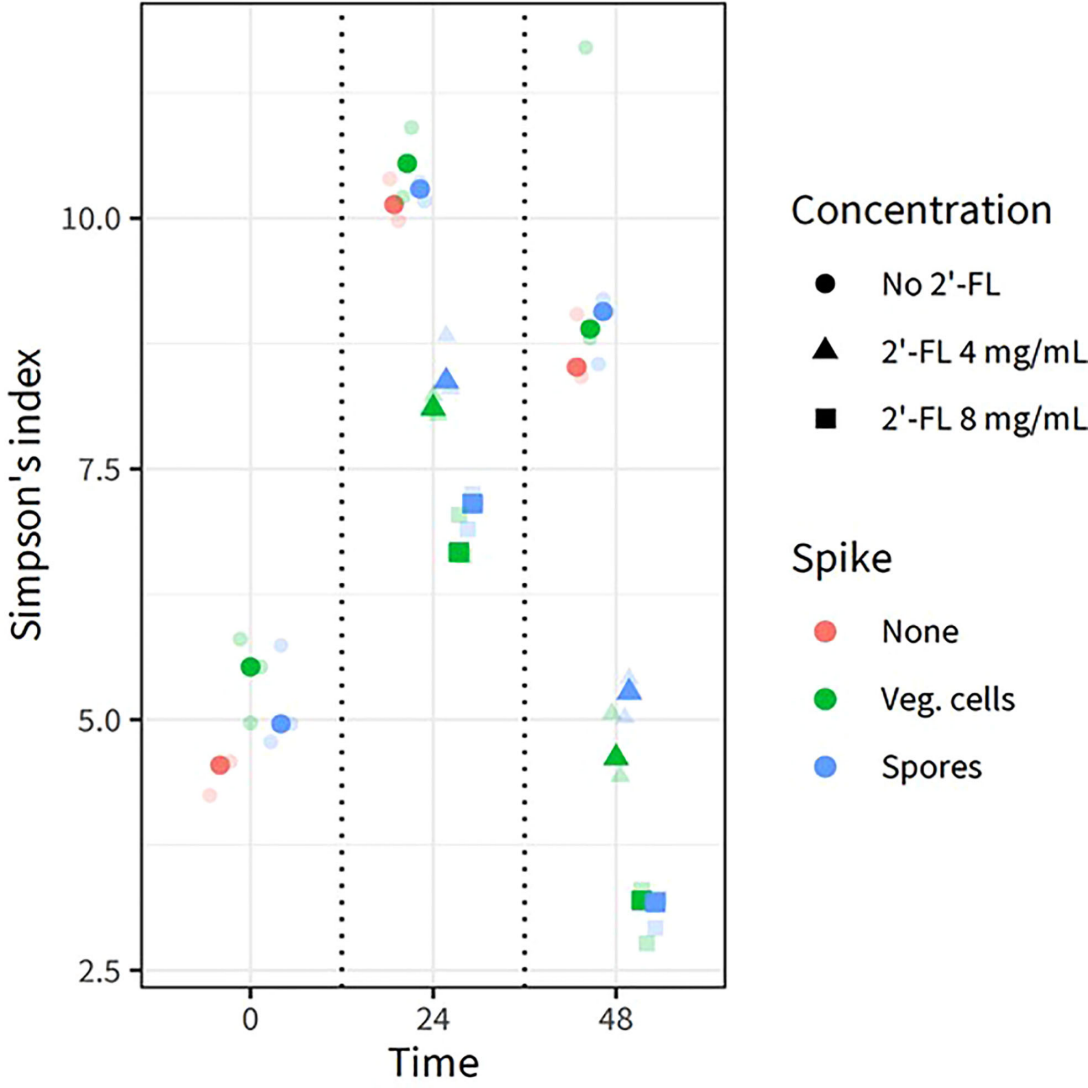
2’FL affects the alpha diversity in the CDi-screen model Alpha diversity derived from 16S rRNA gene sequencing data of *in vitro* experiments is displayed as the Simpson Index (on the y-axis) for all experimental conditions and samples replicates as indicated by the different colored symbols. Each centroid shows its time point of sampling, with each sample shown in lower opacity. The fecal microbial community was analyzed without *C. difficile* (red) as well as with *C. difficile* vegetative cells (green) or spores (blue), and incubated in CDi-SIEM media at pH 6.0, without (circles) and with supplementation of the media with 2’-FL at doses of 4 mg/mL (triangles) and 8 mg/mL (squares), and sampled at t=0 h, 24 h, 48 h (indicated on the x-axis).

We also analyzed the alpha diversity (displayed as Simpson’s index, **Figure 3**) within the samples to investigate if the intrinsic microbial diversity under the test conditions changed through the addition of 2’-FL and/or *C. difficile* vegetative cells and spores. Each centroid shows its time point of sampling, its color representing the absence of *C. difficile*, the presence of vegetative cells or spores, and its shape representing the concentration of 2’-FL. The highest diversity was noted at 24h for all conditions, indicating community restructuring during incubation in the CDi-screen model. Consistent with the PCA, samples with and without *C. difficile* clustered closely in the absence of 2’-FL. Samples without *C. difficile* spores or vegetative cells and without the additional supplementation of 2’-FL displayed the highest Simpson’s index at 24 h and 48 h. Microbial communities derived from samples with *C. difficile* and 2’-FL supplementation displayed a lower Simpson’s index and this effect was more pronounced at a later sampling time and a higher 2’-FL dose. Thus, 2’-FL supplementation of the media leads to a decrease in diversity within the sample, likely by selectively promoting or inhibiting specific taxa within the microbial communities.

**Figure 4:**
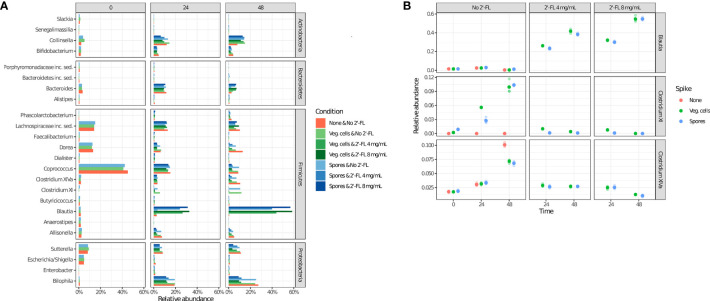
2’-FL changes the relative abundance of specific genera in the CDi-screen over time. Microbial community composition as determined *via* 16S rRNA gene sequencing is displayed as relative abundance at the genus level. The community composition dynamics throughout the incubation were investigated at the time points 0 h, 24 h, and 48 h, as indicated, without or with 2’-FL at a concentration of 4 mg/mL or 8 mg/mL. The absence (red circles) or presence of *C difficile* ATCC 43599 inoculated as vegetative cells (green circles) or spores (blue circles) is shown. **(A)** The relative abundance of the top 25 most abundant genera under all experimental conditions (shown on the right). **(B)** Relative abundances of the selected genera Clostridium cluster XI (which includes *C difficile*), *Blautia*, and *Clostridium* cluster XIVa (extracted from the source data for **Figure 3**).

To assess which genera most likely contribute to the observed effect, we have performed an analysis of the relative abundance of the 25 most abundant microbial genera identified in the current study under all experimental conditions (**Figure 4A**). The results support the finding that microbial community composition is dynamic, even in the absence of *C. difficile* and 2’-FL supplementation, as clear differences in the relative abundance of particular genera are observed (e.g. *Coprococcus, Bacteroides, Clostridium XIVa*)(4 A) at different time points. An increase in the Clostridium cluster XI (to which *C. difficile* belongs) up to a relative abundance of 3-4% after 24 h and approximately 12% after 48 h of incubation in the absence of 2’-FL is consistent with the expansion of *C. difficile* upon inoculation with either spores or vegetative cells under those conditions (**Figure 4A**).

**Figure 5:**
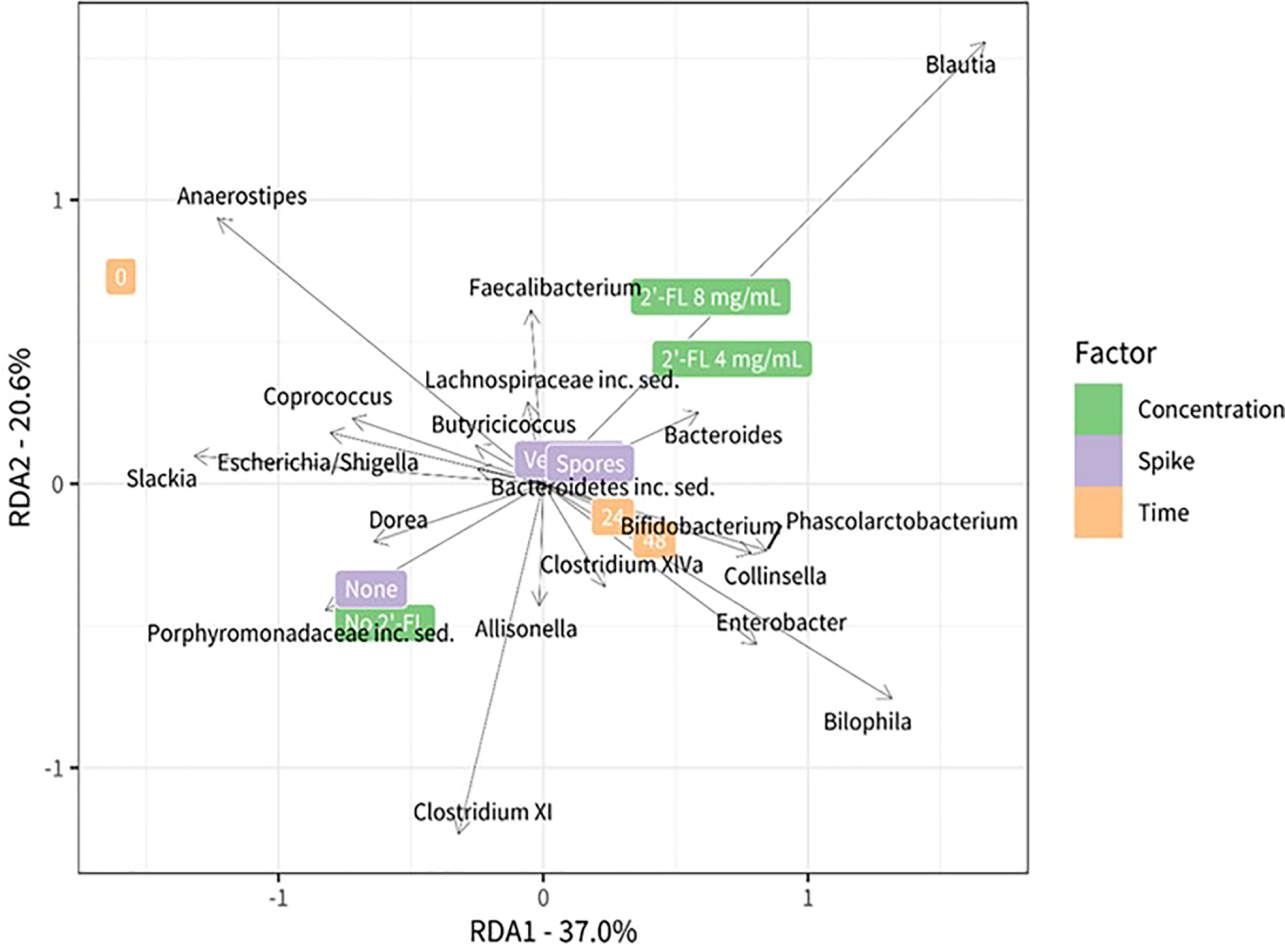
Redundancy analysis plot using center-log transformed data showing the relationship between the test conditions and bacterial genera. Different test conditions are highlighted on the right side of the plot green: concentration 2’-FL, violet: *C. difficile* spike, orange: timepoints. The position in the plot is determined by the microbiota composition in the corresponding condition. The black vectors represent the bacteria that are most associated with the test conditions: their length and orientation are indicative of their relationship with the different test conditions.

The addition of 2’-FL into the CDi-screen was associated with a striking increase in the relative abundance of the genus *Blautia*, and a concomitant decrease in *Clostridium* XI and XIVa (**Figure 4A, B**). The increase in the genus *Blautia* was dose-dependent (**Figure 4A, B**). It resulted in approximately 50% of the relative abundance of *Blautia* after 48 h of incubation at a dose of 8 mg/mL and about 40% of the relative abundance at a 4mg/mL dose. Conversely, we observed progressively lower levels of *Clostridium* cluster XIVa with increasing amounts of 2’-FL, whereas *Clostridium* cluster XI (which includes *C. difficile*) was already reduced to a very low level at 4 mg/mL of the HMO (**Figure 5**).

We further analyzed the contribution of specific, and potentially more lowly abundant, genera using a redundancy analysis (RDA). The RDA analysis includes all taxa that remained after filtering but the plot shows only the top 20 taxa that have the largest contribution to the difference between the conditions. Microbial communities grown without 2’-FL were characterized by the presence and or abundance of the genera *Dorea, Slackia, Escherichia, Shigella*, and genera belonging to the Clostridium XI cluster. The genus *Anaerostipes* was more abundant at time point zero, whereas prolonged incubation time led to an increase in the relative abundance of the genera *Bilophila*, *Enterobacter*, and genera belonging to the *Clostridium* XIVa cluster.

Combined with the insights on the proliferation levels of *C. difficile* gained through qPCR analysis, it is apparent that the growth dynamics of *C. difficile* within a complex microbial community will differ depending on the microbial community composition and related metabolites. The specific community composition is impacted through interventions throughout time as can be seen in **Figure 2**, and differs after 24 and 48 hours. The microbial community samples for the doses of 4mg/ml and 8 mg/ml cluster closer together at 24 h and separate more clearly after 48h of incubation. The dose-dependent reduction of *C. difficile* observed for the 2-’FL in comparison with the no substrate control may hence be indirect (microbiota-related effects). *C. difficile* proliferation may be inhibited depending on the microbial community structure and associated metabolites.

## Discussion

Here, we have shown the adaptation of the i-screen model (Ladirat et al., 2013; Fehlbaum et al., 2018; Schuren et al., 2019), for the study of *C. difficile* over a 48 h period. The CDi-SIEM medium sustains germination, growth, and toxin production of *C. difficile* ATCC 43599 both in pure culture and in a complex microbial community derived from human donor feces. The 2’-FL supplementation at two different doses (4 mg/mL and 8 mg/mL) effectively prevented the proliferation of *C. difficile* in a microbial community. Whereby, the *C. difficile* spore inoculum resulted in slightly lower cell levels compared to the numbers detected in conditions inoculated with vegetative cells, possibly due to a compromised fitness of freshly germinated cells.

The CDi-screen addresses a need for higher throughput experimental models to allow for a cost-efficient analysis of multiple replicates per condition. The experimental set-up of the CDi-screen with sufficient technical replicates for all experimental conditions (n=3) allowed us to extract the dose-dependent statistical significance of the 2’-FL intervention. The inclusion of technical replicates in the CDi-screen results in statistically relevant data output and displays an advantage over other model systems that do not include technical replicates in the experimental design, likely due to high costs, large working volumes, and low experimental throughput (e.g. a recent study by Vigsnaes et al., 2021 (Vigsnaes et al., 2021)). Though in this study we used pooled human fecal material to mimic a complex microbial community as is common in these type of approaches (Aguirre et al., 2014), we note that future application of the CDi-screen model would also allow to investigate differences in the response to therapeutic interventions that are mediated by differences in microbiome composition between hosts.

2’-FL is part of a group of highly diverse non-digestible complex carbohydrates that play a crucial role in creating and maintaining a healthy infant gut microbiota through prebiotic effects and promoting the growth of beneficial bacteria such as *Bifidobacterium* and *Lactobacillus* (Locascio et al., 2007; De Leoz et al., 2015). HMOs may also modulate the gut microbiota of adults (Al-Khafaji et al., 2020; Šuligoj et al., 2020). Upon reaching the intestine, HMOs may confer prebiotic effects, act as anti-adhesives, and bind or interact with toxin binding sites (Bode, 2009; Asadpoor et al., 2021).

In this study, we have seen an increase in *Blautia* when supplementing the CDi-screen with 2’-FL in the presence of *C. difficile*. In addition, we detected a reduction in *C. difficile* in 2’-FL supplemented media and it is tempting to speculate that the observed reduction in *C. difficile* is related to the expansion of *Blautia*. The *Blautia* genus has been recently reviewed as a new functional genus with potential probiotic properties (Liu et al., 2021), *Blautia* has also been associated with alleviating inflammatory diseases and metabolic diseases and for its antibacterial activity against specific microorganisms, e.g., for instance *via* the production of antibiotics. Trypsin-treated nisin-like peptides produced by *Blautia* have been reported to be highly active against two clinical pathogens, *C. perfringens* and *C. difficile* (Hatziioanou et al., 2017). The production of lantibiotics by *Blautia* may play a role in the inhibition of *C. difficile in vitro*. Bacteria such as *Blautia* may also produce metabolites that impact *C. difficile* growth or compete directly for resources. Identifying the specific strain involved in the observed effects would allow furthering the investigation of potential *C. difficile* inhibitory effects.

It is unknown if the expansion of *Blautia* is a direct or indirect effect of the 2’-FL supplementation. The *Blautia* genus plays a role in biotransformation and crosstalk with other intestinal microorganisms (Liu et al., 2021). Compared to common probiotic bacteria such as lactic acid bacteria (LAB), the proportion of genes related to carbohydrate transport and metabolism is relatively small in *Blautia* (6.57%), indicating that it may have a more limited ability to metabolize carbohydrates (Liu et al., 2021). The effect of 2’-FL may therefore be specific, or potentially require cross-feeding with other microbes.

Future work could also consider the addition of inulin to the CDi-screen. Recently, chicory inulin was found to enhance the fermentation of 2′-FL by infant fecal microbiota (Akkerman et al., 2021). Beneficial effects of inulin on the gut microbiota and colonization resistance against *C. difficile* have been described in several studies (Van Nuenen et al., 2003; Koleva et al., 2012; Hryckowian et al., 2018). It would be interesting to investigate if such effects can also be detected for adult and/or elderly microbial communities and increase the inhibitory effects against *C. difficile*.

In this study, we have shown the CDi-screen platform for assessing the effects of interventions at different doses on the proliferation of one *C. difficile* strain *in vitro*. Due to its high throughput, we expect that the CDi-screen offers excellent possibilities to test i) the effects of different substrate mixes and doses that may assist in optimized formulations for (pre)clinical trials; ii) interventions prior, during, or after disturbing the microbiota using antimicrobials, aimed at minimizing the loss of colonization resistance against *C. difficile*; and iii) the effect of compounds on a panel of diverse and clinically relevant *C. difficile* isolates, to exclude strain-specific effects.

## Data availability statement

The datasets presented in this study can be found in online repositories. The names of the repository/repositories and accession number(s) can be found below: https://https://www.ncbi.nlm.nih.gov/sra/PRJNA858224, Biosamples are: SRX16159794 - SRX16159844.

## Author contributions

Study design: JV, MW, AO, FS, MH, LV, EK. Data Analysis: TB, AO, MH, MW, JV. Writing of the Manuscript: MW, JV, WS, PB. All authors contributed to the article and approved the submitted version.

## Funding

The project was funded by Health~Holland (LSH-TKI match project LSHM 18088) as well as the partners DSM (Glycom) and Mercurius Production GmbH.

## Conflict of interest

Author LV was employed by Glycom A/S—DSM Nutritional Products Ltd.

The authors declare that the research was conducted in the absence of any commercial or financial relationships that could be construed as a potential conflict of interest.

## Publisher’s note

All claims expressed in this article are solely those of the authors and do not necessarily represent those of their affiliated organizations, or those of the publisher, the editors and the reviewers. Any product that may be evaluated in this article, or claim that may be made by its manufacturer, is not guaranteed or endorsed by the publisher.
