# Supplementary material for: 2’-Fucosyllactose inhibits proliferation of Clostridioides difficile ATCC 43599 in the CDi-screen, an in vitro model simulating Clostridioides difficile infection
Source: Front Cell Infect Microbiol. 2022 Oct 28;12:991150. doi: 10.3389/fcimb.2022.991150 (PMC9650113; doi:10.3389/fcimb.2022.991150)
Supplement: Supplementary file 1 [file DataSheet_1.pdf]

Supplementary Material

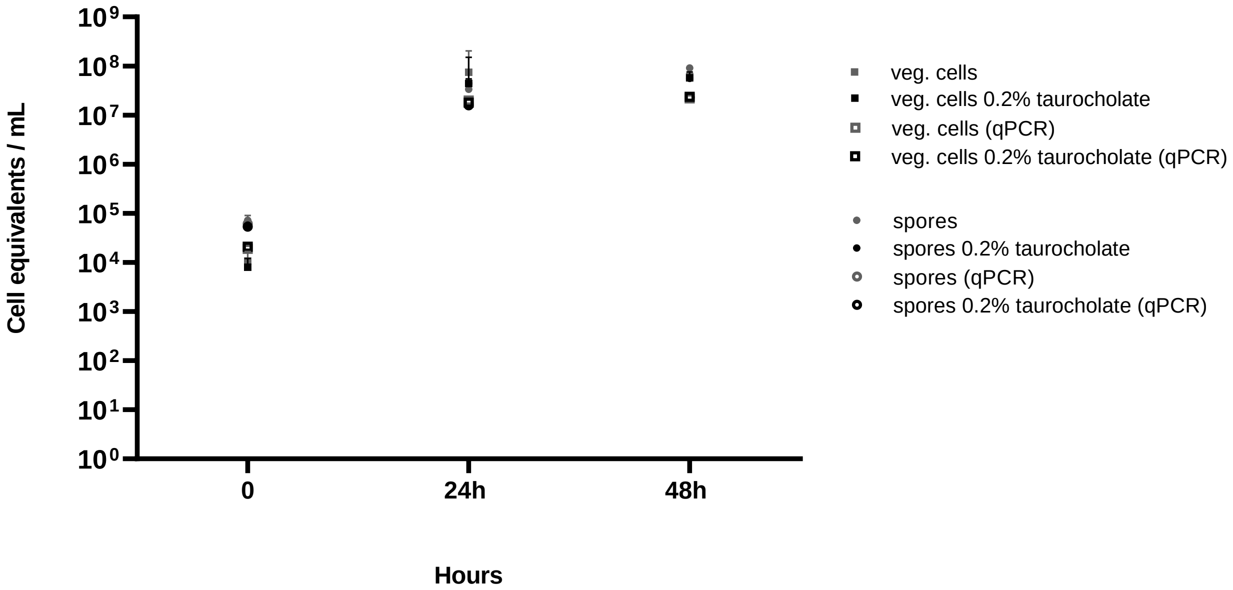

Supplementary Figure 1. Cell proliferation of the *C. difficile* ATCC 34599 strain was followed for conditions inoculated with spores or vegetative cells of the strain as monoculture into the CDi-SIEM media with and without supplementation with 0.2% taurocholate, grown for 48 hours under anaerobic conditions at 37°C. The figure depicts the *C. difficile* cell number equivalents detected via the counting of colony forming units (CFUs) and via *C. difficile* specific (16S rRNA primer- based) qPCR. The average cell number equivalents (n=3) are depicted at the time points 0, 24, 48 hours.
